# NLR Inflammasomes in Viral Infections: From Molecular Mechanisms to Therapeutic Interventions

**DOI:** 10.3390/v18050546

**Published:** 2026-05-08

**Authors:** Shiyuan Hou, Xing Shen, Danni Sun, Yulin An, Yuxuan Zhou, Xing Sun, Shuhan Wang, Xinyue Liu, Mengting Zhu, Shuai Zhao, Ziyu Liu, Xingan Wu, Rongrong Liu

**Affiliations:** 1 Department of Microbiology, School of Basic Medical Sciences, Fourth Military Medical University, Xi’an 710032, China; 2 School of Basic Medical Sciences, Fourth Military Medical University, Xi’an 710032, China

**Keywords:** inflammasome, NLRs, viral pathogenesis, immune evasion, pyroptosis, NLRP3, GSDMD, SARS-CoV-2, therapeutic inhibitors

## Abstract

The innate immune system serves as the primary barrier against viral invasion, utilizing pattern recognition receptors (PRRs) to orchestrate a rapid defense. Among these, the nucleotide-binding domain and leucine-rich repeat (NLR) containing proteins function as central signaling scaffolds, assembling into multiprotein complexes known as inflammasomes. These complexes drive the maturation of pro-inflammatory cytokines IL-1β and IL-18, and initiate gasdermin D (GSDMD)-mediated pyroptosis, a lytic cell death pathway that eliminates intracellular replication niches. This comprehensive review synthesizes the diversified landscape of inflammasome activation during viral infections, extending beyond the canonical NLRP3 inflammasome to include specialized sensors such as NLRP6, NLRP9, NLRP1, NLRP12, and NLRC4. We critically evaluate the evolutionary “arms race” between host defenses and viral pathogens, detailing the sophisticated immune evasion strategies employed by viruses—ranging from the expression of decoy proteins and direct proteolytic cleavage of immune sensors to the manipulation of post-translational modifications (PTMs). Furthermore, we discuss the dual nature of inflammasome activation, which balances protective viral clearance against pathological hyperinflammation, and provide an exhaustive analysis of novel therapeutic strategies, including direct NLR inhibitors and downstream cytokine blockers, currently navigating clinical transition.

## 1. Introduction

**Figure 1:**
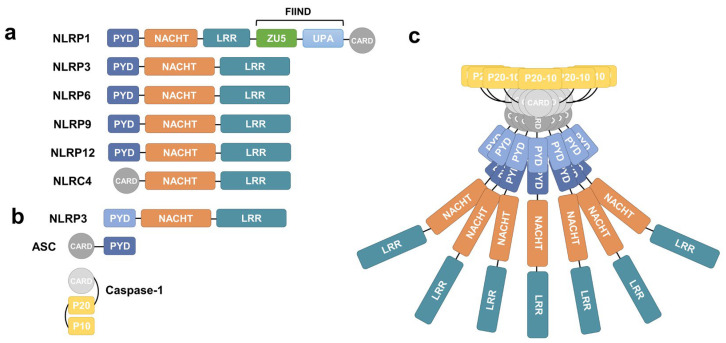
(**a**) Domain architectures of NLRP1, NLRP3, NLRP6, NLRP9, NLRP12, and NLRC4. (**b**) The canonical sensor–adapter–effector mechanistic model of NLRP3 inflammasome assembly. NLRP3 recruits the adapter ASC via PYD–PYD interactions, which in turn engages caspase-1 through CARD–CARD interactions. (**c**) Molecular model of NLRP3 inflammasome oligomerization and activation. NLRP3 aggregates into clusters, wherein the NLRP3 PYD nucleates to form fibrillar filaments. These NLRP3 PYD filaments subsequently recruit ASC PYD, driving its nucleation into ASC PYD filaments. In turn, ASC CARD filaments recruit and nucleate caspase-1 CARD filaments via CARD–CARD interactions. Ultimately, the p20/p10 domains of caspase-1 undergo self-cleavage, activating its proteolytic activity.

Since the seminal conceptualization of the inflammasome two decades ago, these macromolecular complexes have emerged as pivotal executioners of the innate immune response [1]. Inflammasomes comprise three core components: a sensor molecule (typically a nucleotide-binding domain and leucine-rich repeat (NLR) containing protein, also known as a NOD-like receptor), an adaptor protein (the apoptosis-associated speck-like protein containing a caspase recruitment domain, abbreviated as ASC), and an effector protease (caspase-1); these complexes convert pathogen sensing into potent inflammatory outputs [2,3] (Figure 1). While their roles in bacterial defense are well-established, recent breakthroughs have illuminated the critical and complex function of inflammasomes in viral infections [4].

During viral infection, host cells recognize pathogen-associated molecular patterns (PAMPs) or damage-associated molecular patterns (DAMPs) through pattern recognition receptors (PRRs), activating inflammasome complexes to initiate innate immune responses. Inflammasomes assemble sensor proteins, such as NLR family or Absent In Melanoma 2 (AIM2), adaptor protein ASC, and effector protein caspase-1 to mediate caspase-1 activation [5]. Activated caspase-1 cleaves GSDMD, releasing its N-terminal fragment. This fragment binds to phosphoinositides in the plasma membrane and oligomerizes to generate membrane pores of about 12–14 nm in inner diameter, triggering the release of pro-inflammatory cytokines (e.g., interleukin-1β (IL-1β) or IL-18) and inducing pyroptosis [6,7,8]. Different inflammasomes play a central role in anti-infective immunity, inflammation regulation, and cell death pathways by specifically recognizing viral components such as nucleic acids, proteins, or host damage signals [7,9,10].

The dual role of inflammasomes in viral infections—serving as both defense mechanisms and potential contributors to pathology—has come into sharp focus during recent global health crises. A paradigmatic case is the global SARS-CoV-2 pandemic: appropriate activation restricts viral replication via pyroptotic cell death and cytokine-mediated immune recruitment, whereas aberrant or excessive activation leads to systemic immunopathological injury, a phenomenon epitomized by the cytokine storms observed in severe COVID-19 [11,12,13,14,15].

**Figure 2:**
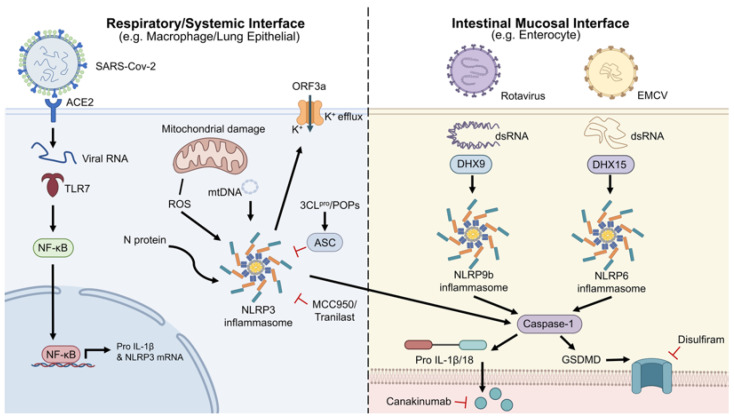
The distinct sensing pathways of NLR inflammasomes at different host–pathogen interfaces. (**Left panel**) Respiratory/systemic interface: SARS-CoV-2 infection triggers NLRP3 activation via a two-step mechanism (priming and activation) involving NF-кB signaling, mitochondrial ROS, and K^+^ efflux. (**Right panel**) Intestinal mucosal interface: NLRP9b detects rotavirus dsRNA via DHX9, while NLRP6 senses viral RNA via DHX15. (Downstream): All sensors recruit ASC and caspase-1 to induce IL-1β/18 secretion and GSDMD-mediated pyroptosis. Evasion and Therapy: Viral evasion strategies (e.g., POPs) and therapeutic targets (e.g., MCC950, disulfiram, canakinumab) are highlighted.

Importantly, the interaction between viruses and inflammasomes is not unilateral. Rather, it represents a dynamic evolutionary arms race in which host cells deploy inflammasome sensors to detect infection, whereas viruses evolve countermeasures to blunt or redirect these responses [16]. In line with the scope of the “Viral Mechanisms of Immune Evasion” Special Issue, this review focuses on the principal NLR inflammasomes implicated in viral infection—including NLRP3, NLRP6, NLRP9, NLRP1, NLRP12, NLRC4—and discusses three interconnected themes: mechanisms of activation, viral immune-evasion strategies, and emerging therapeutic interventions (Figure 2).

## 2. Mechanistic Insights into NLR Activation and Regulation

### 2.1. NLRP3: A Sophisticated Two-Step Activation Model

The NLRP3 inflammasome is the most versatile sensor, responding to a diverse array of viral stimuli. NLRP3 is typically maintained in an autoinhibited state [17]. Its activation typically follows a two-step ‘priming and activation’ model [1,18]: Priming (Signal 1): Viral PAMPs (e.g., ssRNA) trigger Toll-like receptors (TLRs), activating the NF-кB pathway to upregulate the transcription of NLRP3 and pro-IL-1β [19,20]. Activation (Signal 2): A plethora of cellular stress signals trigger the assembly of the complex. These include K^+^ efflux induced by viral viroporins (e.g., SARS-CoV-2 ORF3a, influenza M2), mitochondrial ROS production, and lysosomal rupture [21,22,23,24]. Activation signals induce conformational changes in NLRP3, enabling it to oligomerize and recruit ASC via pyrin–pyrin domain interactions. ASC then recruits pro-caspase-1 through CARD–CARD interactions, together forming the assembled NLRP3 inflammasome [14]. Subsequently, pro-caspase-1 undergoes autocatalytic activation, cleaving pro-IL-1β, pro-IL-18, and GSDMD. The cleaved GSDMD then forms pores in the plasma membrane, releasing IL-1β and IL-18 and inducing pyroptosis [25].

Across these studies, a unifying pattern emerges: distinct viruses engage different proximal triggers, yet most converge on a shared NLRP3 activation architecture consisting of NF-κB-dependent priming, disruption of cellular homeostasis, and ASC-caspase-1 assembly [1,20]. Thus, NLRP3 functions less as a receptor for a single viral ligand than as an integrated hub that senses virus-induced stress. For example, the SARS-CoV-2 N protein directly binds to the C-terminal domain (260–340 aa) of NLRP3, promotes the NLRP3–ASC interaction, facilitates ASC oligomerization, and subsequently activates caspase-1 in a NEK7-independent manner [26]. In HIV-1 infection, viral proteins such as Vpr, gp120, and Tat, together with extracellular ATP release from infected cells and purinergic signaling, drive potassium efflux and calcium influx, thereby promoting NLRP3 inflammasome assembly and caspase-1 activation [27]. After IAV infects host cells, the newly synthesized M2 protein is transported to the trans-Golgi network during viral replication, leading to the dispersion of TGN structure into punctate structures that recruit NLRP3, induce its conformational activation and oligomerization, and promote ASC recruitment and caspase-1 activation [21]. These examples collectively explain why NLRP3 activation can contribute to both antiviral restriction and immunopathology through IL-1β/IL-18 release and pyroptosis [28,29]. The “licensing” of NLRP3 is strictly governed by post-translational modifications (PTMs) [30]. Ubiquitination is essential for the regulation of NLRP3 inflammasome function. After infection with herpes simplex virus 1 (HSV-1), stimulator of interferon genes (STING)—a central molecule in antiviral and inflammatory immune pathways—interacts with NLRP3. This interaction reduces NLRP3 polyubiquitination, improves the localization of NLRP3 in the endoplasmic reticulum, and promotes the activation of the NLRP3 inflammasome [31]. NLRP3 is modified by lysine-63 (K63) ubiquitin chains in hepatocytes and activated via deubiquitination during hepatitis C virus (HCV) infection [32].

Viruses have also evolved multiple mechanisms to evade NLRP3 inflammasome activation. Viruses directly inhibit downstream NLRP3 inflammasome activation through the suppression of both priming and activation steps. A case in point is hepatitis B virus (HBV), which attenuates NLRP3 inflammasome activation and IL-1β secretion via inhibition of the NF-κB signaling pathway and ROS production [33].

Viruses could control the activation status of NLRP3 by manipulating modifications. Epstein–Barr virus (EBV) encodes the G protein-coupled receptor BILF1 and the UFM1 E3 ligase UFL1, both of which are associated with MAVS. MAVS is a key binding site in mitochondria and a driver of NLRP3 oligomerization. BILF1 blocks NLRP3 inflammasome activation by inducing MAVS UFMylation [34]. HIV evades inflammasome activation by activating E3 ubiquitin ligases, which induce NLRP3 degradation in the proteasome [35]. Zika virus (ZIKV) NS1 recruits host USP8 to cleave K11-linked polyubiquitin chains from caspase-1 at Lys134, inhibiting its proteasomal degradation [36]. SARS-CoV-2 papain-like protease exerts a negative regulatory effect on the NLRP3 inflammasome pathway by attenuating the oligomerization and ubiquitination of ASC [37].

### 2.2. Mucosal and Organ-Specific Sensors: NLRP6 and NLRP9

While NLRP3 is ubiquitously expressed in myeloid cells, some other NLRs exhibit restricted tissue distributions, functioning as specialized guardians of mucosal barriers. NLRP6 and NLRP9 synergistically orchestrate intestinal antiviral defense.

NLRP6 plays a crucial role in antiviral defense, particularly in the intestine [38,39,40,41,42]. Importantly, increased NLRP6 expression should be distinguished from bona fide inflammasome activation. In the intestinal mucosa, type I interferons (IFNs), PPARγ agonists, and inflammatory cytokines can increase NLRP6 abundance and thereby create a permissive state for signaling, but these events alone do not demonstrate inflammasome assembly [43,44,45]. At the transcriptional level, NLRP6 expression is induced by peroxisome proliferator-activated receptor γ (PPAR-γ) binding to its promoter region [45] as well as by inflammatory signals such as tumor necrosis factor-α (TNF-α) and viral stimuli [40]. A second layer of stimulation is then provided by microbial or viral ligands. Mechanistically, NLRP6 utilizes its C-terminal leucine-rich repeat (LRR) domain to respond to bacterial products such as lipoteichoic acid (LTA) and lipopolysaccharide (LPS) [46,47] and it cooperates with the RNA helicase DEAH-box helicase 15 (DHX15) to sense viral RNA and recruit MAVS for type I/III IFN induction [40,48] In this context, microbiota-derived metabolites such as taurine, histamine, and spermine should be considered modulators of NLRP6 signaling rather than uniformly direct activators [40,48]. Structurally, full-length NLRP6 can assemble into filamentous structures with the PYD at the core and the NBD-LRR regions at the periphery, a configuration that may facilitate signal transmission to ASC [49]. Upon activation, NLRP6 oligomerizes through PYD–PYD interactions, recruits ASC, and activates caspase-1 or caspase-11 [49]. This assembly results in the cleavage of pro-IL-1β and pro-IL-18 into mature cytokines and GSDMD, inducing pyroptosis, while also modulating NF-κB and MAPK signaling pathways in a cell- and context-specific manner [50,51]. Negative regulation is achieved through deubiquitination by Cyld, which cleaves K63-linked ubiquitination of NLRP6, inhibiting its interaction with ASC and preventing excessive inflammation [52]. In the present review, the discussion of NLRP6 is therefore confined mainly to intestinal antiviral defense, where its inflammasome and non-canonical interferon-organizing functions are best supported.

The viral infection activation mechanism of NLRP9 involves its role as an inflammasome sensor that restricts rotavirus replication in intestinal epithelial cells. Specifically, upon rotavirus infection, the RNA helicase DHX9 binds to viral double-stranded RNA (dsRNA) and forms a complex with NLRP9b (the predominant isoform expressed in the murine ileum), though the exact interaction mechanism remains unclear [53,54]. This complex triggers NLRP9b inflammasome assembly by recruiting the adaptor protein ASC via homotypic PYD–PYD interactions, which subsequently activates caspase-1 through CARD–CARD interactions [53]. Active caspase-1 cleaves pro-IL-18 into its mature form and activates GSDMD, leading to pyroptosis—a proinflammatory cell death that eliminates infected cells [53]. This rapid cell death prematurely aborts the viral replication cycle and expels infected enterocytes, preventing viral dissemination. While IL-18 secretion is induced, studies in mice suggest that GSDMD-dependent pyroptosis, rather than IL-18, is the primary mechanism for controlling rotavirus [53,55]. Structurally, the human NLRP9 PYD exists as a monomer and cannot self-polymerize like other NLRPs, such as NLRP3, implying that full-length NLRP9 may require co-factors DHX9-RNA or other domains to overcome autoinhibition for inflammasome activation [56,57].

Taken together, NLRP6 and NLRP9b appear to constitute a spatially organized antiviral surveillance system in the intestine. NLRP6 preferentially binds long double-stranded RNA (dsRNA) and is enriched in the duodenum (proximal small intestine), whereas NLRP9b exhibits stronger binding to short dsRNA and is primarily expressed in the ileum (distal small intestine) [44]. This division of labor provides a more coherent explanation for their complementary roles in enteric viral defense than treating the two sensors as isolated observations.

### 2.3. NLRP1: Predominant Inflammasome in Barrier Cells

Human NLRP1 was the first identified inflammasome-forming sensor, highly expressed in epithelial tissues, which has been confirmed to be present in the stomach, small intestine, colon, lungs, endometrium, and skin keratinocytes [58,59,60,61,62]. All in all, NLRP1 is the major inflammasome in barrier cells [63]. Unlike other NLR family proteins, NLRP1 has some distinct structural domains: its CARD domain is located at the C-terminus, rather than the N-terminus as in other NLR molecules, and the LRR is connected to the CARD via a function-to-find domain (FIIND) [64,65,66,67]. FIIND contains two subdomains: ZU5 and UPA. This domain auto-processes NLRP1 into two polypeptide chains, allowing NLRP1 to exist in an inactive state as two non-covalently bound polypeptides—the N-terminal domain and a fragment containing the C-terminal CARD, where cytosolic dipeptidyl peptidase 9 (DPP9) sequesters the C terminus of NLRP1 to repress inflammasome activation [64,65,66,68].

NLRP1 can be activated by multiple stimuli including viral dsRNA, cleavage by viral proteases, ribotoxic stress, and inhibition of dipeptidyl peptidases 8 and 9 (DPP8/9) [69]. NLRP1 can directly interact with dsRNA and subsequently acquires adenosine triphosphatase (ATPase) activity, activating the inflammasome pathway [58]. NLRP1 senses intron-containing RNA (icRNA) in HIV-1-infected macrophages and microglia cells, directly activating itself [70]. Ribotoxic stress like UVB exposure causes RNA damage and activates stress-activated protein kinases like p38 and ZAKα. ZAKα and p38 hyperphosphorylate the serine residues in the linker region of NLRP1 between the PYD and NACHT domains, thereby releasing the UPA-CARD domain to form a functional inflammasome [71]. During α-virus infection, P38 directly phosphorylates NLRP1, inducing the ubiquitination of PYD domain, the N-terminal degradation of NLRP1, and inflammasome nucleation [72]. The open reading frame 45 protein (ORF45) of Kaposi’s sarcoma-associated herpesvirus (KSHV) binds to Linker1, dissociates UPA from the Linker1–UPA complex, and induces the release of the C-terminal domain of hNLRP1 to assemble the inflammasome [73]. The non-structural protein of severe fever with thrombocytopenia syndrome virus (SFTSV) binds to FIIND and degrades DPP8/9 to disrupt the NLRP1 inhibitory complex, thereby inducing NLRP1 activation [74].

Another activation mechanism of NLRP1 was first discovered in mouse Nlrp1 [75]. The lethal factor (LF) protease subunit of anthrax lethal toxin (LeTx) specifically cleaves the N-terminus of mouse NLRP1B. Subsequently, through a protein quality control mechanism called the “N-end rule” pathway, the NLRP1B N-terminal domain is targeted for proteasomal degradation, which gradually degrades the N-terminal domain, releases the bioactive C-terminal CARD-containing fragment of NLRP1B, and initiates downstream inflammasome activation [76,77,78]. Moreover, the N-terminal domain itself is not required for NLRP1 activation but functions in pathogen sensing [76]. In humans, various pathogen proteases have been found to possess the ability to cleave and activate NLRP1 [58,76,79]. Human rhinovirus 3C protease (HRV-3Cpro) specifically cleaves NLRP1 between p.Q130 and G131, leading to inflammasome activation mediated by the C-terminal fragment of NLRP1 (amino acids 1213 to 1474) [79]. NLRP1 acts as a sensor for SARS-CoV-2 infection in pulmonary epithelial cells; the SARS-CoV-2 3CL protease (3CLpro) cleaves NLRP1 at the Q333 site, triggering inflammasome assembly and cell death and restricting the production of infectious viral particles [79]. This activation capability is referred to as viral “tripwire” immunity [63].

Although NLRP1 is activated through multiple pathways by sensing viral nucleic acids and proteases, viruses have evolved complex mechanisms to evade NLRP1 activation. The early gene F1L of the vaccinia virus acts upstream of ZAKα, blocking dsRNA- and ribotoxic stress-dependent NLRP1 activation [80]. HSV-1 produces the viral E3 ubiquitin ligase infected cell protein 0 (ICP0), which prevents proteasomal degradation of the autoinhibitory NLRP1 N-terminal fragment, thereby preventing inflammasome formation [81].

### 2.4. NLRP12: A Context-Dependent Modulator of Inflammation and Antiviral Responses

NLRP12 is a cytoplasmic innate immune sensor that functions predominantly as a negative regulator of inflammatory signaling, especially through inhibition of the NF-κB and MAPK pathways, although it can participate in inflammasome assembly under selected conditions. NLRP12 contains a PYD, a central nucleotide-binding domain (NBD), and a C-terminal LRR region, enabling it to respond to PAMPs or DAMPs [82]. Mechanistically, NLRP12 suppresses canonical NF-κB signaling by interacting with hyperphosphorylated interleukin-1 receptor-associated kinase-1 (IRAK1) [83], and it restrains non-canonical NF-κB signaling by promoting the proteasomal degradation of NF-κB-inducing kinase (NIK) through TRAF3-dependent mechanisms [84,85]. Under some circumstances, NLRP12 can also recruit ASC, activate caspase-1, and promote the maturation of IL-1β and IL-18 [86,87]. NLRP12 activation is triggered by various stimuli, including bacterial infections, viral components, and cellular stress, which can lead to its oligomerization and downstream effects on immune responses such as cytokine production and cell migration [88,89]. Viral evidence remains comparatively limited and is not predominantly intestinal. One antiviral example is the inhibition of dengue virus (DENV) replication through the interaction of NLRP12 with HSP90 via its NACHT domain, which promotes type I interferon and ISG production [90].

Viruses may also exploit NLRP12 to promote pathological inflammation. SARS-CoV-2 3CLpro cleaves NLRP12 at two sites (residues 238 and 938), disrupting its structural integrity, abolishing its restraint on NF-κB signaling and potentially perturbing NLRP3 inflammasome assembly, thereby contributing to cytokine overproduction in severe COVID-19 [91].

### 2.5. NLRC4: A Non-Canonical Inflammasome Bridging Bacterial Sensing and Emerging Antiviral Immunity

The NLRC4 inflammasome activation mechanism is activated after cytosolic ligand detection by NAIP family proteins, which recognize stimuli such as flagellin or type III secretion system (T3SS) [92,93]. Ligand-bound NAIP undergoes a conformational change that enables interaction with inactive NLRC4 monomers [94,95], relieving ADP-mediated autoinhibition and initiating wheel-like oligomerization of NLRC4 [94,95,96]. The assembled complex recruits caspase-1 directly or via ASC, leading to the maturation of IL-1β/IL-18 and cleavage of GSDMD [97,98,99]. Regulatory mechanisms include transcriptional control by interferon regulatory factor 8 (IRF8) and post-translational modifications such as the phosphorylation of NLRC4 at serine 533 by kinases like protein kinase Cδ (PKCδ) and leucine-rich repeat kinase 2 (LRRK2) [100,101,102].

In viral infection, NLRC4 regulates lung dendritic-cell function and CD4+ T-cell responses during IAV infection [103]. During HSV-1 infection, it enhances the K63-linked polyubiquitination of TBK1 by promoting the interaction between TBK1 and the E3 ubiquitin ligase CBL, thereby strengthening cGAS–STING–TBK1 signaling [104]. In the intestine, current antiviral evidence is more indirect and is best exemplified by the TLR5/NLRC4-dependent induction of IL-22 and IL-18 during rotavirus control rather than by a clearly established virus-specific NLRC4 sensing mechanism [105].

Overall, NLRP12 and NLRC4 should not be interpreted as intestine-specific antiviral inflammasomes in the same way as NLRP6 and NLRP9b. Rather, current evidence suggests broader immunoregulatory roles with only limited or context-dependent intestinal antiviral relevance.

## 3. Viral Mechanisms of Inflammasome Evasion

To establish persistent infection and replicate efficiently, viruses have evolved a diverse arsenal of strategies to dismantle inflammasome signaling. These evasion mechanisms, summarized in Table 1, operate at multiple checkpoints, from signal interference to the direct degradation of key structural components (Table 1).

### 3.1. Direct Targeting of Inflammasome Components

Proteolytic cleavage is a hallmark of viral evasion. Emerging evidence highlights that the SARS-CoV-2 3CLpro directly cleaves NLRP12, disrupting its ability to regulate inflammation and potentially destabilizing the inflammasome complex [91]. Enterovirus 3C and 2A proteases have been reported to cleave NLRP3, physically severing the link between sensing and effector functions [106,107]. HIV activates E3 ubiquitin ligases to induce NLRP3 degradation in the proteasome [35]. Enteric viruses utilize TRIM29 to promote K48-linked ubiquitination, leading to the degradation of NLRP6 and NLRP9b [108]. HSV-1 ICP0 prevents proteasomal degradation of the autoinhibitory NLRP1 N-terminal fragment [81].

### 3.2. Disruption of Assembly and PTMs

Viruses manipulate PTMs to keep sensors in an inactive state. The IAV NS1 protein interacts with NLRP3 to suppress its ubiquitination and oligomerization, thereby arresting the “licensing” step required for activation [109]. Additionally, poxviruses encode pyrin-only proteins (POPs) that act as “molecular decoys” [110]. These viral proteins bind to ASC via PYD–PYD interactions, preventing the recruitment of caspase-1 and the formation of functional inflammasome specks [110,111].

### 3.3. Targeting Upstream Pathways

Viruses inhibit inflammasome activation by targeting upstream pathways. To selectively degrade mitochondrial membrane cargos and block NLRP3 inflammasome activation, EBV BILF1 utilizes the UFMylation pathway to direct MAVS to MDV trafficking and lysosomal degradation [34]. The GP5 protein of porcine reproductive and respiratory syndrome virus (PRRSV) promotes endoplasmic reticulum (ER)–mitochondria contact to enhance mitochondrial Ca^2+^ uptake from the ER, thereby triggering the release of mitochondrial reactive oxygen species (mROS). Elevated mROS induces autophagy, which alleviates NLRP3 inflammasome activation to optimize viral replication [112]. African swine fever virus (ASFV) pB318L negatively regulates the NF-κB signaling pathway, blocking the initiation stage of NLRP3 inflammasome activation [113]. The early gene F1L of the vaccinia virus acts upstream of ZAKα, blocking dsRNA- and ribotoxic stress-dependent NLRP1 activation [80].

**Table 1:** Inflammasomes implicated in viral infection and viral evasion mechanisms.

Sensor	Primary Activation Signal	Representative Viruses	Viral Evasion Mechanism	Ref.
NLRP3	K+ efflux, ROS, PTM licensing	IAV, HIV, Enterovirus, EBV, PRRSV, ASFV	IAV NS1 downregulates NLRP3 inflammasome activation by targeting NLRP3 as well as NF-κB; HIV activates E3 ubiquitin ligases; enterovirus 3C and 2A proteases cleave NLRP3; EBV BILF1 utilizes UFMylation to induce lysosomal degradation; PRRSV GP5 triggers ROS release; and ASFV pB318L negatively regulates the NF-κB signaling pathway.	[34,35,106,112,113,114]
NLRP6	dsRNA (via DHX15)	Enteric RNA viruses	Enteric viruses utilize TRIM29 to promote K48-linked ubiquitination, leading to the degradation of NLRP6 and NLRP9b.	[108]
NLRP9b	dsRNA (via DHX9)			
NLRP1	dsRNA, viral protease, ribotoxic stress	Vaccinia virus, HSV-1	Vaccinia virus F1L acts upstream of ZAKα; HSV-1 E3 ubiquitin ligase ICP0.	[80,81]
NLRC4	Phosphorylation, NAIP crosstalk	HSV-1, IAV	Mechanism undefined.	[103,115]

## 4. Therapeutic Strategies: From Bench to Bedside

The identification of inflammasomes as key regulators of immune responses to viral infections has paved the way for novel therapeutic strategies. Because direct pharmacological tools remain most developed for NLRP3, the current translational landscape is weighted toward this sensor. Nevertheless, therapeutic intervention can also be organized according to target level—upstream priming pathways, inflammasome assembly, downstream pore formation, or terminal cytokine signaling—thereby extending relevance beyond a single NLR family member (Table 2).

### 4.1. Inflammasome Inhibitors

The initial step in inflammasome activation involves signaling cascades that trigger the production of pro-inflammatory cytokines. Strategies aimed at inhibiting inflammasome initiation focus on suppressing the expression of inflammasome-related proteins and associated signaling molecules. For instance, dexmedetomidine and rapamycin have been shown to inhibit the NLR inflammasome pathway by decreasing NF-κB expression, which is crucial for the transcription of pro-IL-1β and pro-IL-18 [116,117]. In SARS-CoV-infected mice, the NF-κB antagonists CAPE, resveratrol, Bay11-7082, and parthenolide improved survival and reduced proinflammatory cytokine levels in the lungs [118]. Sulforaphane has been shown to suppress the expression of the NLRP3 gene and thereby inhibit inflammasome activation at the transcriptional level [119]. CS-82 targets LRRK2 kinase activity to suppress NLRC4 inflammasome activation [120]. Thus, although many agents were first characterized in relation to NLRP3, several act at shared upstream nodes and may have broader anti-inflammasome potential.

Direct interference with inflammasome activation can be achieved through multiple mechanisms. One approach involves blocking ion channels and modulating cellular ion homeostasis, which is often disrupted during viral infections. Sulfasalazine, an anti-inflammatory drug, has been reported to inhibit NLRP3 inflammasome activation by interfering with potassium efflux [121]. Nonsteroidal anti-inflammatory drugs (NSAIDs) have also demonstrated the ability to inhibit NLRP3 inflammasomes by modulating chloride transport across membranes [122]. These strategies effectively disrupt the signal 2 pathway of inflammasome activation, thereby mitigating excessive inflammatory responses.

Targeting the aggregation and assembly of inflammasome complexes is another promising therapeutic approach [123,124,125]. MCC950, a diarylsulfonylurea small molecule, is a selective inhibitor of the NLRP3 pathway and directly interacts with the Walker B motif within the NLRP3 NACHT domain, thereby inhibiting NLRP3 activation and inflammasome formation [123]. Melatonin suppresses NLRP3 complex formation and may improve clinical outcomes in inflammatory viral disease settings including COVID-19 [126,127]. Glibenclamide interferes with the NEK7–NLRP3 axis [128], whereas stress granules and AKT-dependent DDX3X phosphorylation can restrain NLRP3 activation during IAV infection [129]. Tranilast, a clinically used anti-allergic drug and tryptophan-metabolite analog, directly binds to NLRP3, inhibiting its oligomerization and assembly [130]. Colchicine disrupts microtubules and thereby indirectly hinders NLRP3 oligomerization [124]. In addition, BRCC3-related regulation of the NLRP6–DHX15–MAVS axis highlights that therapeutic modulation is not limited to NLRP3 alone [131].

Additionally, direct inhibition of caspase-1 protease activity can suppress downstream effector functions. For instance, parthenolide and CPG 15d-PGJ2 have been shown to block the activation of NLRP1 and NLRP3 inflammasomes by inhibiting caspase-1 protease activity [132,133]. RKIP, arsenic trioxide, and sodium arsenite could both inhibit CASP-1 activation and IL-1β secretion [134,135].

### 4.2. Targeting Downstream Signaling Pathways Such as GSDMD

Targeting downstream molecules of inflammasome activation is crucial for modulating cytokine release and cellular pyroptosis. Representative GSDMD-directed compounds include necrosulfonamide and disulfiram, which target Cys191/192, suppress GSDMD-N oligomerization, and prevent pore formation [136,137]. The inhibitor GI-Y1 binds to GSDMD-Arg7 and blocks the membrane oligomerization of GSDMD-N [138], whereas BAY 11-7082 covalently modifies Cys191 and inhibits pore formation [139]. By limiting GSDMD activity, these drugs can reduce intracellular viral replication niches and excessive cytokine release, thereby mitigating inflammatory damage. Disulfiram, with its long history of clinical use, is especially notable for its translational potential [137,140]. However, GSDMD inhibition must be interpreted in a context-dependent manner: in systemic infections such as COVID-19 it may mitigate lung injury [141], whereas in enteric infections, GSDMD-mediated extrusion of infected cells is a protective mechanism [142,143].

### 4.3. Cytokine Antagonism

Inflammasome activation leads to the release of pro-inflammatory cytokines, which can become harmful in excess. Therapeutic strategies targeting these cytokines, such as IL-1β, IL-18, and IL-6, aim to mitigate cytokine storms. Drugs like canakinumab, sarilumab, and tocilizumab have been developed to inhibit these cytokines, demonstrating efficacy in treating viral infections associated with cytokine release syndrome (CRS). Canakinumab, a human anti-IL-1β monoclonal antibody, has been successfully used to treat inflammasome-related disorders [144,145]. Similarly, tocilizumab and sarilumab, which target IL-6, have shown promise in preventing lung damage and inflammation in COVID-19 patients [146,147].

### 4.4. Novel Treatment Strategies

Nanomedicine strategies have been explored to suppress the NLR inflammasomes/GSDMD signaling pathway [148]. For instance, a biomimetic nanoparticle platform (PDA@M), comprising a polydopamine core coated with a macrophage membrane, has been developed to inhibit the NLRP3-caspase-1 signal [149]. A carbonic anhydrase IX (CAIX)-targeted rhenium(I) photosensitizer (CA-Re) has been shown to induce GSDMD-mediated pyroptosis [150]. Conversely, phospholipid-coated sodium citrate nanoparticles (PSCT NPs) activate both the caspase-1-GSDMD and caspase-8-GSDMC pathways [151]. Beyond nanomaterials, prospective gene-based strategies—such as RNA interference or genome-editing approaches directed against NLRP3, ASC, caspase-1, or GSDMD—are conceptually attractive, although antiviral applications remain largely preclinical at present.

Hydrogels, as emerging biomaterials, have also been applied in NLRP3-targeted therapies. Researchers have engineered an injectable, pH-responsive hydrogel (MH@ZIF-8/CS/β-GP) that effectively suppresses the NLRP3/caspase-1/IL-1β signaling pathway and scavenges ROS [152].

**Table 2:** Clinical status of inflammasome-targeting candidates.

Candidate	Mechanism of Action	Disease Focus	Development Stage	Ref.
MCC950	Direct NLRP3 NACHT Inhibitor	Sepsis	Pre-clinical	[153]
Dexmedetomidine	NF-кB inhibitor	COVID-19	Phase IV	[154]
Sulforaphane	NLRP3 expression suppression	HIV	Phase IV	[155]
Resveratrol	NF-кB antagonists	COVID-19	Phase II	[156]
Tranilast	Direct NLRP3 inhibitor	Severe COVID-19	Phase II/III	[157]
Colchicine	Blocks NLRP3 inflammasome oligomerization	COVID-19	Phase II	[158]
Disulfiram	GSDMD pore blocker	COVID-19	Phase II	[159]
Canakinumab	IL-1β neutralizing mAb	COVID-19-induced pneumonia	Phase III	[160]
Tocilizumab	IL-6 receptor antagonist	COVID-19	FDA approved	[161]

## 5. Conclusions

Current antiviral treatments focus predominantly on viral replication inhibitors, but few therapies target the host’s immune system, particularly inflammasomes. Given the critical role of inflammasomes in balancing antiviral defense and immunopathology, exploring inflammasomes as potential therapeutic targets, new antiviral strategies could emerge. For instance, inhibiting excessive inflammasome activation could mitigate virus-induced cytokine storms, a phenomenon observed in severe cases of infections such as SARS-CoV-2 [162]. Additionally, the specific targeting of inflammasome pathways like NLRP6 could provide targeted treatment approaches, especially in tissue-specific viral infections. However, these mechanisms remain underexplored, and further research is needed to identify precise inhibitors that can modulate inflammasome activity without compromising overall immune function.

Compared with conventional antiviral therapies, inflammasome-targeted treatments offer several advantages. Traditional antiviral drugs often face challenges such as resistance development and limited efficacy across different viral strains. In contrast, therapies targeting inflammasome pathways could circumvent these limitations by modulating the host’s immune response, making them broadly applicable across a range of viral infections. However, translating inflammasome inhibition into clinical practice poses its own set of challenges. One major concern is the potential for immunosuppression, which could render patients vulnerable to opportunistic infections [9]. Further research is needed to develop inflammasome inhibitors that are selective enough to prevent excessive inflammation without impairing immune defenses.

Although significant advancements have been made in understanding the role of inflammasomes in viral infections, key gaps remain. The exact mechanisms through which different viral pathogens activate specific inflammasomes, such as NLRP3, remain incompletely understood. Furthermore, how inflammasomes contribute to the progression of viral infections versus their protective roles is still an unresolved issue. A classic example is DENV infection, where DENV infection leads to robust inflammasome activation, and there is a direct association between inflammasome activation and the severity of dengue fever: inflammasome-induced IL-1β plays a central role in tissue damage and vascular leakage [163]. On the other hand, inflammasome activation may exert a protective role during DENV infection—mice lacking caspase-1/11 are more susceptible to DENV infection than their wild-type littermates with intact caspase-1/11 function [164]. This suggests that the timing and magnitude of inflammasome activation may be key factors in determining whether the activation is protective or detrimental to the host [164].

Another critical area that requires further attention is the differential activation of inflammasomes in various tissues, which may account for disease-specific pathology. A deeper understanding of the regulatory mechanisms that control inflammasome activity during viral infections could uncover novel therapeutic approaches. For example, in sepsis, NLRP3 inflammasome activation exhibits marked organ-specificity [165]. In alveolar macrophages, increased NLRP3-dependent release of IL-1β and IL-18 enhances microvascular permeability and induces alveolar epithelial cell death. In the kidney, NLRP3 inflammasome activation promotes the pyroptosis of tubular cells and amplifies local inflammation through the secretion of IL-1β and IL-18, forming a self-perpetuating cycle of injury. In cardiomyocytes and cardiac resident macrophages, NLRP3 inflammasome activation contributes to the development of sepsis-induced myocardial dysfunction (SIMD) through multiple mechanisms: IL-1β and IL-18 directly impair contractile function by impairing L-type calcium channel function and sarcoplasmic reticulum Ca^2+^-ATPase function, thereby impairing calcium handling capacity [165].

Inflammasomes stand at the crossroads of antiviral defense and immunopathology. While significant progress has been made, future research must address critical gaps including defining the “trigger threshold” for pathological inflammation and developing tissue-specific inhibitors. As viruses continue to evolve evasion mechanisms, understanding the molecular arms race at the inflammasome interface will be key to designing the next generation of antiviral immunotherapies.

## Data Availability

No new data were created or analyzed in this study. Data sharing is not applicable to this article.

## Abbreviations

The following abbreviations are used in this manuscript:

ASC	Apoptosis-associated speck-like protein containing a CARD
ASFV	African swine fever virus
ATP	Adenosine triphosphate
BHRF1	BamHI fragment H rightward open reading frame 1
CARD	Caspase recruitment domain
CRS	Cytokine release syndrome
DAMPs	Damage-associated molecular patterns
DENV	Dengue virus
DHX9	DEAH-box helicase 9
EBV	Epstein–Barr virus
ER	Endoplasmic reticulum
FADD	Fas-associated protein with death domain
FBXL2	F-box and leucine-rich repeat protein 2
GSDMD	Gasdermin D
HCMV	Human cytomegalovirus
HSV-1	Herpes simplex virus 1
IAV	Influenza A virus
IECs	Intestinal epithelial cells
IFNs	Interferons
IL-1β	Interleukin-1β
IRF3	Interferon regulatory factor 3
JNK1	c-Jun N-terminal kinase 1
LLPS	Liquid–liquid phase separation
MAVS	Mitochondrial antiviral-signaling protein
mROS	Mitochondrial reactive oxygen species
NAIP	NLR Family Apoptosis Inhibitory Protein
NLR	Nucleotide-binding domain and leucine-rich repeat
NLRP3	NLR family pyrin domain containing 3
NLRC4	NLR family CARD domain containing 4
NS1	Non-structural protein 1
NSAIDs	Nonsteroidal anti-inflammatory drugs
ORF3a	Open reading frame 3a
PAMPs	Pathogen-associated molecular patterns
PKCδ	Protein kinase C delta
POPs	Pyrin-only proteins
PRRs	Pattern recognition receptors
PRRSV	Porcine reproductive and respiratory syndrome virus
PTMs	Post-translational modifications
PYD	Pyrin domain
ROS	Reactive oxygen species
SARS-CoV-2	Severe acute respiratory syndrome coronavirus 2
TLRs	Toll-like receptors
NF-кB	Nuclear factor kappa-B
